# *RMCProfile7*: reverse Monte Carlo for multiphase systems

**DOI:** 10.1107/S1600576724004175

**Published:** 2024-07-31

**Authors:** Wojciech A. Sławiński, Christopher J. Kerr, Yuanpeng Zhang, Helen Y. Playford, Martin T. Dove, Anthony E. Phillips, Matthew G. Tucker

**Affiliations:** a Faculty of Chemistry University of Warsaw Pasteura 1 02-093Warsaw Poland; b ISIS Facility STFC Rutherford Appleton Laboratory DidcotOX11 0QX United Kingdom; c Department of Chemistry, University of Cambridge, Lensfield Road, CambridgeCB2 1EW, United Kingdom; d Neutron Scattering Division Oak Ridge National Laboratory,Oak Ridge TN37831 USA; e Institute of Atomic and Molecular Physics Sichuan University,Chengdu610065 Sichuan People’s Republic of China; f School of Mechanical Engineering Dongguan University of Technology 1st Daxue Road, Songshan Lake Dongguan523000 Guangdong People’s Republic of China; g School of Physical and Chemical Sciences, Queen Mary University of London, Mile End Road, LondonE1 4NS, United Kingdom; DESY, Hamburg, Germany

**Keywords:** *RMCProfile*, reverse Monte Carlo, big-box modelling, computer programs

## Abstract

This work introduces a completely rewritten version of the program *RMCProfile* (version 7), big-box, reverse Monte Carlo modelling software for analysis of total scattering data. The major new feature of *RMCProfile7* is the ability to refine multiple phases simultaneously, which is relevant for many current research areas such as energy materials, catalysis and engineering. Other new features include improved support for molecular potentials and rigid-body refinements, as well as multiple different data sets. An empirical resolution correction and calculation of the pair distribution function as a back-Fourier transform are now also available. *RMCProfile7* is freely available for download at https://rmcprofile.ornl.gov/.

## Abstract 2

This paper presents the new *RMCProfile7* program for reverse Monte Carlo big-box modelling of multiple-phase systems.

## 1. Introduction

Total scattering data are now widely used to examine structures of polycrystalline materials as they provide information about both the long-range, average crystal structure and local atomic arrangements (Keen, 2020 ▸; Dove & Li, 2022 ▸). This perspective is often a vital part of understanding structure–property relationships. As the total scattering technique has grown in popularity, the samples studied and the experiments themselves have grown in complexity and the need for sophisticated analysis software has concurrently increased.

There are two main approaches one can use to construct an atomistic structural model based on total scattering data:

(i) Small box, where a small crystallographic unit cell is used to describe the structure of the whole crystal. In this approach the real-space pair distribution function (PDF) can be calculated up to any interatomic distance range by assuming that all neighbouring unit cells are exactly identical. The atomic structure representing different length scales can be fitted using different regions of the PDF, thereby giving a view of both local and average atomic structure. This method has been implemented in several computer programs like *PDFGui* (Farrow *et al.*, 2007 ▸), *DiffPy-CMI* (Juhás *et al.*, 2015 ▸), *TOPAS* (Coelho, 2018 ▸) and *Discus* simulation software (Proffen & Neder, 1997 ▸).

(ii) Big box, where a large atomistic model of the structure containing many unit cells is fitted to the PDF and other experimental data. The real-space PDF can be calculated only up to half of the shortest supercell dimension. This approach allows for more sophisticated models of the structure including local atom displacements, short-range ordering on local or medium scale *etc.* Big-box modelling has been implemented in *RMCProfile* (Tucker *et al.*, 2007 ▸), *RMC++* (Gereben *et al.*, 2007 ▸) and *RMC-POT* (Gereben & Pusztai, 2012 ▸).

The reverse Monte Carlo (RMC) technique is an example of a big-box modelling technique (McGreevy & Pusztai, 1988 ▸) whereby the positions of a large ensemble (or configuration) of atoms are adjusted using a Metropolis Monte Carlo algorithm in order to minimize the disagreement between calculated scattering functions and experimental scattering data. While the RMC method was originally developed for the analysis of liquids and amorphous materials, and traditionally only the total scattering pattern and/or the PDF were used to guide the refinement, the Bragg diffraction pattern is an invaluable restraint for the average structure of crystalline materials, and its explicit inclusion in an RMC refinement was the primary purpose of the original *RMCProfile*. Therefore, disordered crystalline materials are an ideal use case from *RMCProfile* (Owen *et al.*, 2024 ▸; Li *et al.*, 2022 ▸; Terban & Billinge, 2022 ▸; Levin *et al.*, 2021 ▸; Dove & Li, 2022 ▸; Dove *et al.*, 2020 ▸; Nygård *et al.*, 2020 ▸, 2021 ▸; Sławiński *et al.*, 2019 ▸).

Many important functional materials, from battery materials and supported catalysts to engineering components, have mixed phases, composed of multiple crystalline phases or a mixture of crystalline and amorphous phases. Naturally, the desire to fully understand those structures means to do so not only in terms of their average structures but also in terms of their local-scale order/disorder or local atomic arrangement. Unfortunately, up to now, it was not possible to use the RMC method, as only a single atomic configuration could be refined using *RMCProfile6* (Tucker *et al.*, 2007 ▸) including special tools for the refinement of nanoparticles (Zhang *et al.*, 2019 ▸).

Refining multiple phases in this way will clearly be essential if each phase has important features in its local structure. Including minority phases will also improve the quality of the majority model, even when the minority phases are not especially interesting in their own right. This is because, in a single-phase refinement against data with contributions from several phases, features due to the minority phases will be ‘fitted’ by distorting the majority phase configuration, unless constraints or restraints prevent this. Incorporating multiple phases into the refinement is thus a natural and effective way of preventing the RMC algorithm from sampling these unphysical regions of configuration space. However, the complexity of an RMC refinement will significantly increase with the number of phases involved.

Even in a single-phase RMC refinement, the large number of parameters typically gives a very low formal data-to-parameter ratio (McGreevy, 2001 ▸). In the language of traditional regression methods, this means that, rather than yielding a single best-fit value of each parameter, refinement instead reveals necessary correlations between these parameters. Physically, such correlations will correspond predominantly to local interactions between atoms, of exactly the sort that PDF analysis aims to identify. Alternatively, using the language of Markov chain Monte Carlo sampling, an RMC-refined model can be described as a sample from the distribution of atomic configurations consistent with the observed data (or, more formally, a sample drawn with probability proportional to the likelihood of it producing the data). Again, analysing a collection of these samples will reveal correlations indicative of the local structure.

Moving to a multiphase refinement, the number of parameters increases without a corresponding increase in the number of data points. Although, as argued above, the absolute number of parameters is not inherently a problem, this decrease in the data-to-parameter ratio must still be taken seriously. This can be ameliorated, for instance, by using multiple data sets, and by incorporating relevant constraints and restraints to maintain the integrity of the structural models. On the other hand, it cannot be tempered by reducing the configuration size of minority phases. Regardless of the phase fraction, the configuration size of each phase must be large enough to allow the PDF to be calculated to the same maximum distance as the experimental PDF data. A hybrid approach where nuisance phases are modelled using ‘small-box’ methods with fewer parameters is conceivable but is not currently implemented in *RMCProfile*.

In order to calculate the PDF for a multiphase system, more complex formulae are required in comparison with a single-phase case as described by Keen (2001 ▸). The full derivation of functions implemented in *RMCProfile7* for multiphase systems has recently been published (Sławiński, 2018 ▸). The two main representations of PDFs for multiphase systems can be expressed in terms of fractional PDFs as the total radial distribution function,
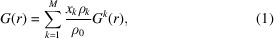
and the differential correlation function,
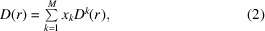
where *G*^*k*^(*r*) and *D*^*k*^(*r*) are PDF functions for individual phase *k* (defined below), *x*_*k*_ is the molar fraction of phase *k*, ρ_*k*_ is the number density of phase *k* and 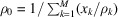 is the overall number density of the multiphase sample.

In the case of neutron PDFs, for each phase *k*, *G*^*k*^(*r*) and *D*^*k*^(*r*) can be written as a function of partial radial distribution functions *g*_*ij*_(*r*) as 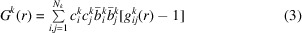
and 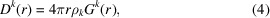
where 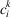 is the proportion of species *i* in phase *k* and 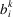 is the coherent neutron scattering length of that species. As *RMCProfile6* and all preceding versions were hardwired for one configuration, it was considered necessary to rewrite the Fortran-based code of *RMCProfile* to allow for the development of multiple-phase refinements and the inclusion of new constraints, as well as to modernize and improve the structure of the code itself. In the next section of this paper the new program, *RMCProfile7*, will be introduced and its major features described, and in the final section some example re­finements will be shown to demonstrate its use. *RMCProfile7* is freely available for download at https://rmcprofile.ornl.gov/.

## 2. New features in *RMCProfile7*

### 2.1. Summary of *RMCProfile6* capabilities

In order to present the variety of new functionalities in *RMCProfile7*, we will first list the most important capabilities of the already well known version of the program *RMCProfile6* (Tucker *et al.*, 2007 ▸) (*RMCProfile* capabilities available in versions 6 and 7 are shown in bold):

(i) **Fitting neutron and X-ray total scattering data (real and reciprocal space)**.

(ii) **Fitting a Bragg data set using instrumental parameters from the *GSAS* (Larson & Von Dreele, 2004 ▸)** or *TOPAS* (Coelho, 2018 ▸) software.

(iii) **Resolution correction in simple form (convolution with a fixed-width Gaussian)** or by using a convolution matrix as obtained from the *TOPAS* software.

(iv) **Constraints and restraints available: distance window, minimum distances, interatomic potentials (distance and angle), bond valence sum, tails**, coordination constraints.

(v) **Swaps between different atomic positions**.

(vi) Magnetic structure modelling in reciprocal space.

(vii) Fitting EXAFS.

(viii) 3D diffuse scattering (neutron, X-ray and electron).

### 2.2. Summary of *RMCProfile7* capabilities

*RMCProfile7* covers most of the functionalities available in *RMCProfile6* shown in bold above but also adds many new features, as listed below. More detailed descriptions will be given in the sections below.

(i) Fitting multiple configurations for multiphase systems.

(ii) Fitting multiple Bragg data sets using instrumental parameters from the *GSAS* (Larson & Von Dreele, 2004 ▸) or *GSAS-II* (Toby & Von Dreele, 2013 ▸) software.

(iii) Calculation of PDFs as a back-Fourier transform of total scattering data.

(iv) Extended list of interatomic potentials available: distance (harmonic, Morse), angles (harmonic, dihedral, inversion), planar.

(v) Rigid body–molecular type moves (translation, rotation and swap).

(vi) Swaps for atom to atom, atom to molecule, and molecule to molecule.

(vii) Atom type description including isotopes, charges and Wyckoff positions.

### 2.3. Multiple configurations

The main new feature of *RMCProfile7* is the ability to include more than one atomic configuration (or box of atoms) in the refinement. This means that, if the sample consists of multiple phases, separate configurations for each phase can be created and refined simultaneously. There is no hard-coded limit on the number of configurations, although it is important to consider whether there is enough information in the available data sets to constrain the refinement of several configurations. As shown in Section 3.1, the reliability of the structural information obtained from the minority phase strongly depends on the system investigated and specific circumstances such as the separation of Bragg peaks in the diffraction pattern.

This method assumes that only intra-phase and no inter-phase interactions need to be accounted for when modelling the data. In the case of inter-phase being a significant contributor to the overall diffraction data, one could consider an extra configuration (treating it as a separate phase) consisting of the inter-phase and carefully weighting all phases present. This is a reasonable assumption for many systems where the interface regions make up only a small fraction of the overall sample, but it may be inappropriate for some, including highly nanostructured materials, intergrowths or nanosized precipitates in alloys. However, in such a case a selection of multiple boxes as representatives of different sample fragments can also be refined using *RMCProfile7*. Alternatively, it is possible to build a really large configuration consisting of all components of the sample in a single box. However, this approach could still lead to unreasonable computing time.

To carry out a multiple-phase refinement in *RMCProfile7* the molar fraction of each phase must be provided as input and cannot (at present) be refined. For a mixture of crystalline phases the phase fractions as determined by Rietveld refinement can be used, whereas for a crystalline/amorphous mixture the fractions will need to be determined by other means, such as density, or if necessary through trial and error.

### 2.4. Multiple data sets

Multiple neutron and/or X-ray PDFs and total scattering functions are already supported in *RMCProfile6*. *RMCProfile7* now adds support for multiple neutron and/or X-ray Bragg data sets. Each data set is fully independent and allows the input of different user-defined ranges and weighting, and as such the user has full control over the way in which their data drive the final refinement. All data sets and all restraints (bond valence sum, potentials and tails) are individually weighted either by the user or by a built-in automated weighting scheme as described by Zhang *et al.* (2020 ▸).

Additionally, for the Bragg data set, a shifted Chebyshev polynomial background function (defined as background function type 1 in the *GSAS* and *GSAS-II* software) can now be refined as the RMC refinement proceeds. A scale factor can also be refined. This option can be useful especially in the case when instrumental and Bragg-peak line-shape parameters are obtained from the Le Bail refinement type. All of this may improve the quality of refinements in certain cases, such as when it is difficult to differentiate the Bragg peaks from broad diffuse signal, but should be used with caution.

The core of an *RMCProfile7* refinement is, of course, the inclusion of one or more total scattering data sets. There are a number of different representations of both real- and reciprocal-space data sets (Keen, 2001 ▸; Peterson *et al.*, 2021 ▸), and in order to make the use of *RMCProfile7* as convenient as possible, most of the commonly used representations are supported. Table 1 ▸ lists and defines these functions, and provides the keywords required to use them in the program. In order to help users transform experimental data sets between different representations, *RMCProfile7* can internally recalculate them into a requested representation.

### 2.5. Simple resolution correction

It is well understood that broadening of diffraction peaks as a result of instrumental resolution or size/strain in the sample leads to an *r*-dependent damping of the PDF signal. If the broadening is severe, the resultant damping can significantly reduce the usable *r* range of the refinement and prevent the program from finding a solution that produces an acceptable fit to all competing data sets (usually PDF versus the Bragg data, depending on the weighting scheme). On the other hand, for the Bragg diffraction data sets, Bragg-peak line-shape parameters are copied from the Rietveld refinement program and not changed/refined in *RMCProfile7*; furthermore, these do not affect real-space PDF or reciprocal-space *F*(*Q*) data sets. To improve this situation, a simple resolution correction has been added to *RMCProfile7*. In real space, an exponential correction is applied as 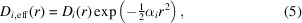
similarly to the 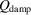 parameter in *PDFGui* (Farrow *et al.*, 2007 ▸), where the resolution correction parameter α_*i*_ can be defined for each phase *i* separately. The value of the α_*i*_ parameter is also set separately for X-ray and neutron data, since the peak profile broadening can be significantly different for the two types of experiments. The corrected 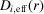 is later Fourier transformed into reciprocal space. The application of this resolution correction has no effect on the Bragg data sets. This form of resolution correction assumes that the reciprocal-space resolution of a data set is roughly constant with *Q* and does not take into account the true *Q*-dependent broadening exhibited by neutron or X-ray data. Nevertheless, it is often sufficient to greatly improve the agreement between observed and calculated data and allow the model to capture both average and local structure more accurately. More accurate *Q*-dependent reciprocal-space data resolution correction will be implemented in future versions of the program, as described by Zhang *et al.* (2020 ▸) ▸.

### 2.6. Real-space PDF calculation as a back-Fourier transform

In the case of neutron scattering, the PDF calculation formalism is straightforward, as described by Keen (2001 ▸). This is because the neutron scattering length *b* is invariant with scattering vector magnitude *Q*. In the case of X-ray scattering, the atomic form factor *f*(*Q*) shows strong damping with *Q* and the *Q* dependence differs for different elements. In most analysis programs, like the commonly used *PDFGui* (Farrow *et al.*, 2007 ▸), a simplified method (here we call it ‘histogram based’) is used. It neglects the fact that the atomic scattering factor *f*(*Q*) damps differently with *Q* for each element. This histogram-based calculation method gives reasonable results but only in the case when the material consists of elements with similar atomic numbers (Dove & Li, 2022 ▸).

A further significant improvement implemented in *RMCProfile7* is to calculate real-space data as a back-Fourier transform of reciprocal-space data. Although this idea, which originated from Masson & Thomas (2013 ▸), has already been used for powder diffraction data and PDF calculation (Neder & Proffen, 2020 ▸), it has been implemented for big-box modelling for the first time in *RMCProfile6* (in the most recent version) and *RMCProfile7*.

First we will focus on the X-ray-based PDF. As described above, the decay of the atomic form factor *f*(*Q*) is different for different elements.

**Figure 1:**
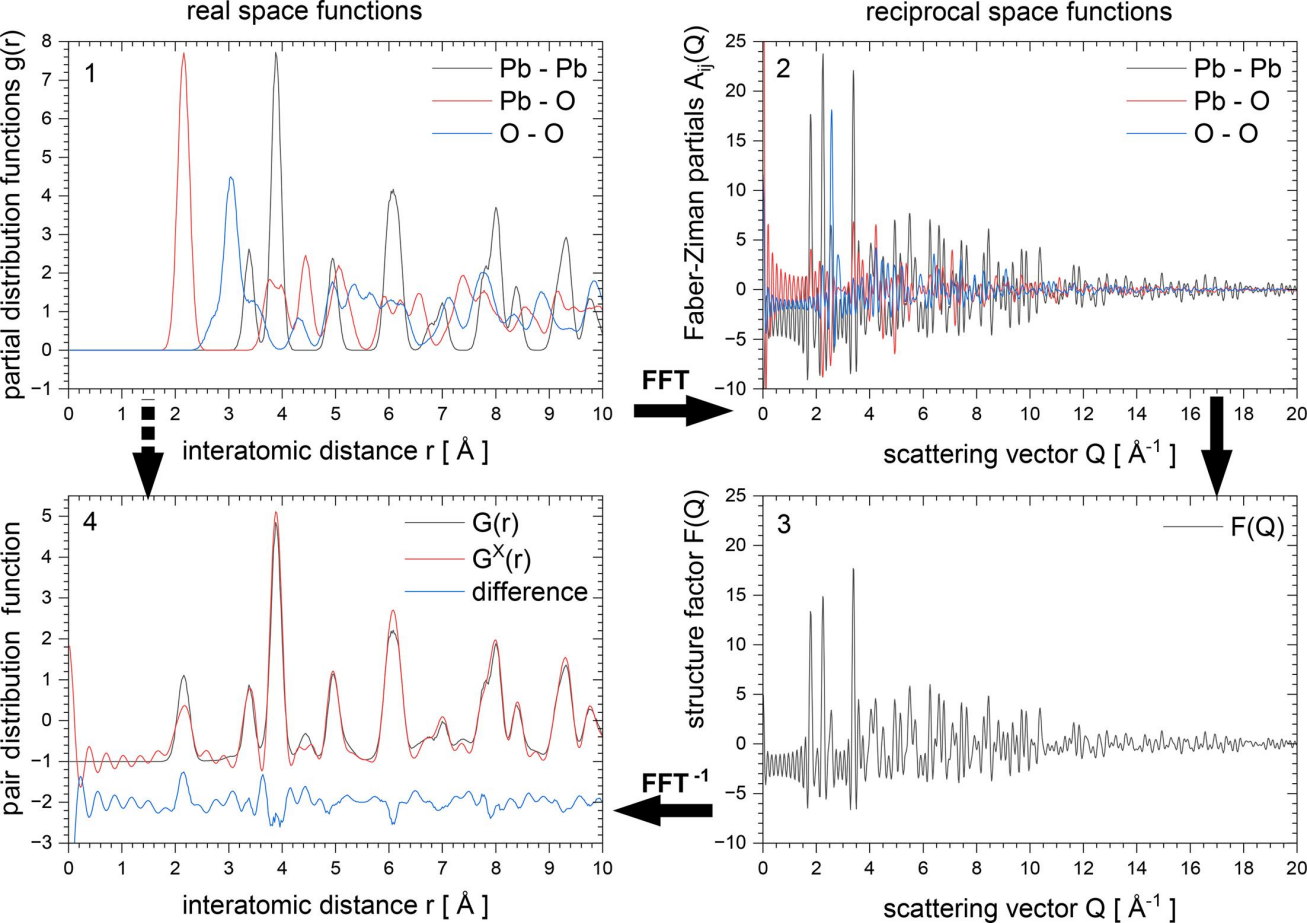
Real-space (panels 1 and 4) and reciprocal-space (panels 2 and 3) functions showing consecutive steps 1, 2, 3 and 4 to calculate the real-space PDF as a back-Fourier transform of scattering factor *F*(*Q*) indicated by solid black arrows. The histogram-based method is shown by the black dashed arrow (directly from step 1 to 4).

Fig. 1 ▸ illustrates the calculation of real-space data as a back-Fourier transform (in the case of an X-ray-based PDF) using the PbO_2_ structure as an example case. The structural model has been taken from Fabrykiewicz *et al.* (2021 ▸). Room-temperature structure parameters, including isotropic dis­placement parameters 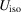, have been used. Panels 1, 2, 3 and 4 show consecutive steps of the calculation:

1: calculation of partial functions (in real space).

1 → 2: Fourier transform of each partial separately into reciprocal space.

2: Faber–Ziman partials (in reciprocal space).

2 → 3: multiplication of Faber–Ziman partials by atomic form factors and summation.

3: final overall structure factor *F*(*Q*).

3 → 4: back-Fourier transform of *F*(*Q*) into the real-space PDF.

4: final real-space PDF *G*^X^(*r*) in comparison with traditional *G*(*r*).

1 → 4: histogram-based way of calculating the X-ray PDF assuming a constant scattering factor.

### 2.7. Constraints and restraints

As already mentioned, in order for an RMC refinement to produce meaningful, chemically and physically reasonable structural models, it is essential to supply as many data sets as possible, as well as to use appropriate constraints and restraints. In this context we use the standard crystallographic convention that constraints are considered to be hard boundaries that cannot be violated and restraints to be soft limitations that are not forbidden, but where violations worsen the goodness of fit or energy penalty.

There are two main constraints available in *RMCProfile7* (as in version 6): minimum distance and distance window. The minimum distance constraint is the familiar ‘hard-sphere cutoff’ which prevents any two atoms of particular type from approaching one another more closely than some user-defined distance. The distance window constraint, as the name implies, defines a window of acceptable distances for a given atom pair, preventing them from getting too close but also preventing them from drifting too far apart. It is particularly useful for maintaining network connectivity. In both cases, the program will reject any move which would violate either constraint.

Two main restraints can be used in *RMCProfile7*. The first is the bond valence sum (BVS) which is defined and used in the same way as in version 6 (Norberg *et al.*, 2009 ▸). This restraint requires the input of suitable bond valence parameters and effectively disfavours moves which cause the calculated BVS for an atom type to move away from the desired value.

The second restraint is that of interatomic potentials. In the case of disordered molecular crystals, it is usually worth sep­arating intra- and intermolecular distances. The intra­molecular distances are often quite well known, as is the geometry of the molecule itself. Therefore, it might be worth restricting individual atom moves to conserve intramolecular distances and geometry by using empirical potentials. On the other hand, the intermolecular arrangement of molecules is usually the most interesting part of the structure refinement. Unfortunately, those two types of interatomic distances often overlap, and so applying intramolecular restraints in the form of interatomic potentials allowed the effects of intermolecular distances to be separated out.

In version 6 of *RMCProfile* there is some support for bond stretching and bond bending potentials, but this functionality has been greatly extended in version 7. Table 2 ▸ lists the available potential types and their *RMCProfile7* keywords.

**Figure 2:**
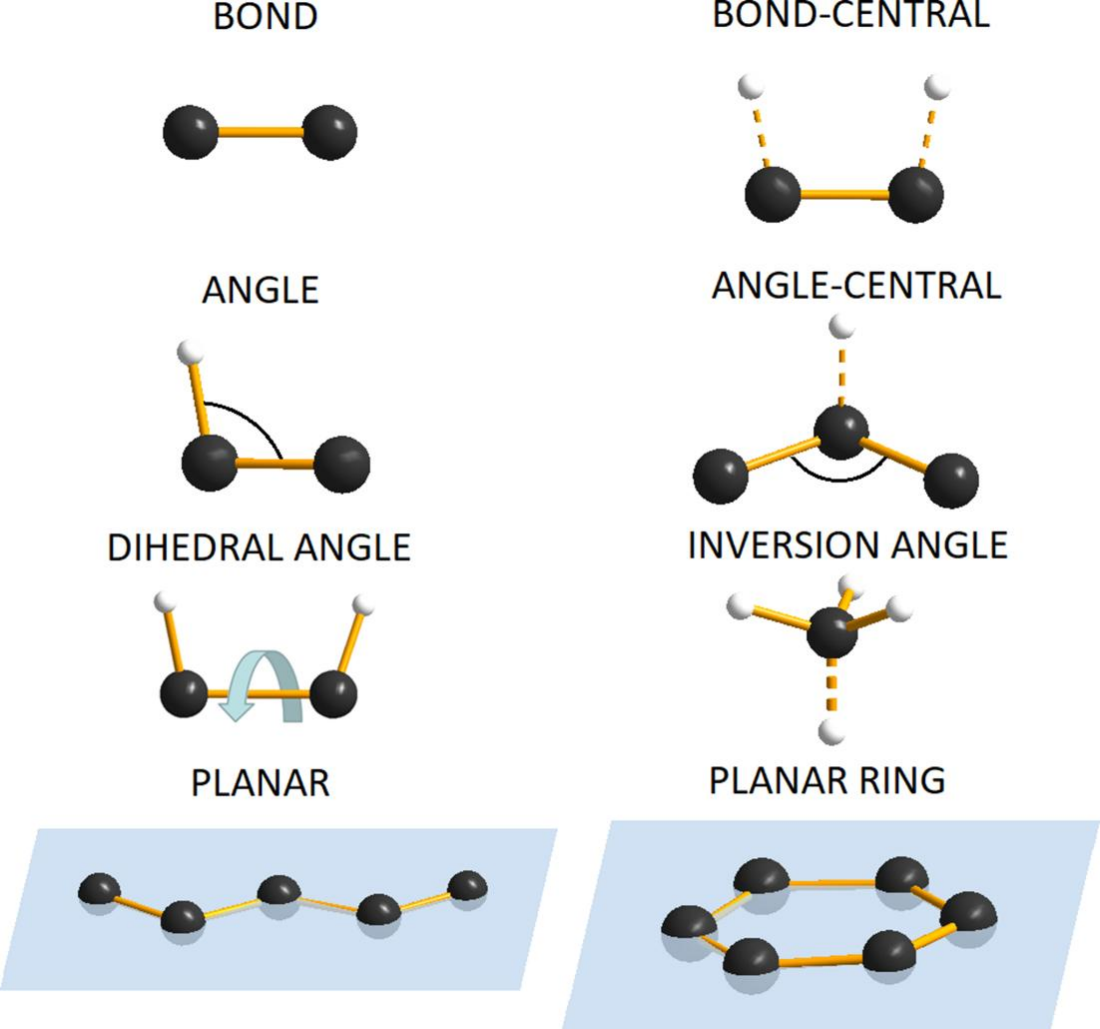
Pictorial examples of different types of potentials available in *RMCProfile7*.

Pictorial examples of the different types of potential are given in Fig. 2 ▸. The use of potentials in *RMCProfile7* requires a supplementary file which contains a list of potentials to be applied separately for each configuration. Multiple potentials can be used simultaneously.

### 2.8. Rigid bodies, molecules

Apart from a multiphase refinement, another significant improvement – molecular type move – has been implemented in *RMCProfile7*. Since many materials contain molecules or molecular ions, this type of move increases the chance of the system to conserve the chemically reasonable shape of rigid-body units.

Rigid bodies are groups of atoms in a structure whose position relative to one another is well defined or well known, such as molecules or coordination polyhedra. During the refinement they can move (translate, rotate and/or swap) as single units. The positional parameters of the constituent atoms are directly determined with respect to the rigid-body origin. In *RMCProfile7* the first atom in the molecule definition is set as its origin. The use of rigid bodies can simplify structural refinements and reduce the number of independent parameters; it is well established in both single-crystal (Scheringer, 1966 ▸) and powder diffraction (Pawley, 1980 ▸; Dinnebier, 1999 ▸).

As determined in Section 3.5 it can also significantly reduce the number of required moves in order to obtain a good model-to-data agreement. Therefore the overall computing time can be significantly decreased.

The ability to define rigid units is a new feature of *RMCProfile7*. As this technique is particularly applicable to molecules, the keyword used in the code is MOLECULE, but it can in fact be any arbitrary group of atoms. To allow for full integration with the other features of the program, each molecule can be translated, rotated and/or swapped with other atoms or other molecules. Currently, molecules can be rotated about an axis defined as a direction in either real or reciprocal space, or about a vector connecting two selected atoms within the molecule, or using Euler angles. An example of using molecular type moves is presented in Section 3.5. This example clearly shows how the use of rigid-body moves can decrease the computing time of the refinement.

### 2.9. Compatibility

As a result of the extensive changes to the code and the introduction of the multiphase capabilities, *RMCProfile7* will not be backwards compatible with input files from earlier versions of *RMCProfile*. However, the overall style and format are very similar. Therefore, users should not encounter much difficulty with converting their files to the new format. Input data files and atomic configuration file formats have been slightly changed. Particularly in the configuration file, a more detailed atom type description is now available. Atomic configurations in rmc7 file format can be created using the *RMCCreate* auxiliary program (formerly known as *data2config*), which is bundled with *RMCProfile7* when downloaded (Dove & Rigg, 2013 ▸).

In particular, a more general description of atom type used in the configuration file is now implemented. For example, in order to code lithium atom isotope 7 with a positive charge 1+, the following description should be used: 7Li1+ (if omitted, the natural abundance and uncharged atom is assumed). In the rmc7 configuration file this atom would be coded as (the header line is added to describe the meaning of all numbers in each line)
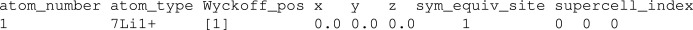


A guide to all of the keywords available in *RMCProfile7* is available at https://rmcprofile.ornl.gov/.

### 2.10. Bragg data set input files

Bragg diffraction reflects the average crystal structure of a material. This is why, in order to obtain a (physically and chemically) reasonable atomistic model of a material, the local atom arrangement as averaged over multiple unit cells has to match its average picture. Therefore we include Bragg diffraction as part of the suite of experimental data, as discussed previously by Tucker *et al.* (2007 ▸). *RMCProfile7* can use several Bragg data sets (X-ray and/or neutron) in contrast to *RMCProfile6*. We currently support inputs from the *GSAS* (Larson & Von Dreele, 2004 ▸), *GSAS-II* (Toby & Von Dreele, 2013 ▸) and *TOPAS* (Coelho, 2018 ▸) Rietveld refinement programs. The exact procedure for data from each of these codes is described in the *RMCProfile7* tutorials.

## 3. Examples

### 3.1. Rutile and anatase mixture – a multiphase sample

Our first example presents the main new capability of the *RMCProfile7* program which is the ability to refine multiple phases at the same time. The experimental data were obtained using the Polaris diffractometer at the ISIS Facility, Rutherford Appleton Laboratory, UK. A physical mixture of 50 mol% of the rutile and anatase phases of TiO_2_ was loaded in a cylindrical thin-walled vanadium can of diameter 6 mm and measured at room temperature for 3 h to obtain reasonable counting statistics for a total scattering study. An initial Rietveld refinement in the *GSAS* software (Larson & Von Dreele, 2004 ▸) confirmed the two-component mixed-phase sample.

**Figure 3:**
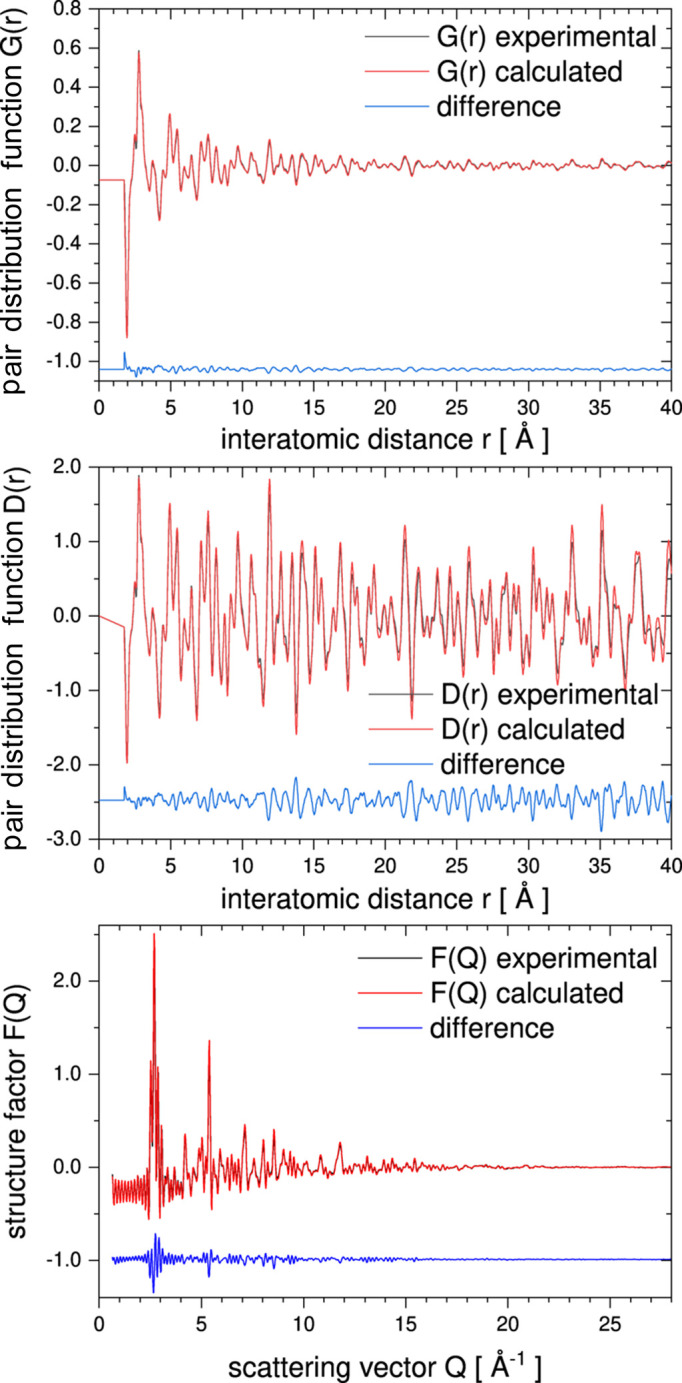
*RMCProfile7* refinements of a physical mixture 50 mol% of rutile and anatase. Experimental data, *RMCProfile7* calculation and difference curves of the PDF *G*(*r*) (top), *D*(*r*) (middle) and structure factor *F*(*Q*) (bottom).

**Figure 5:**
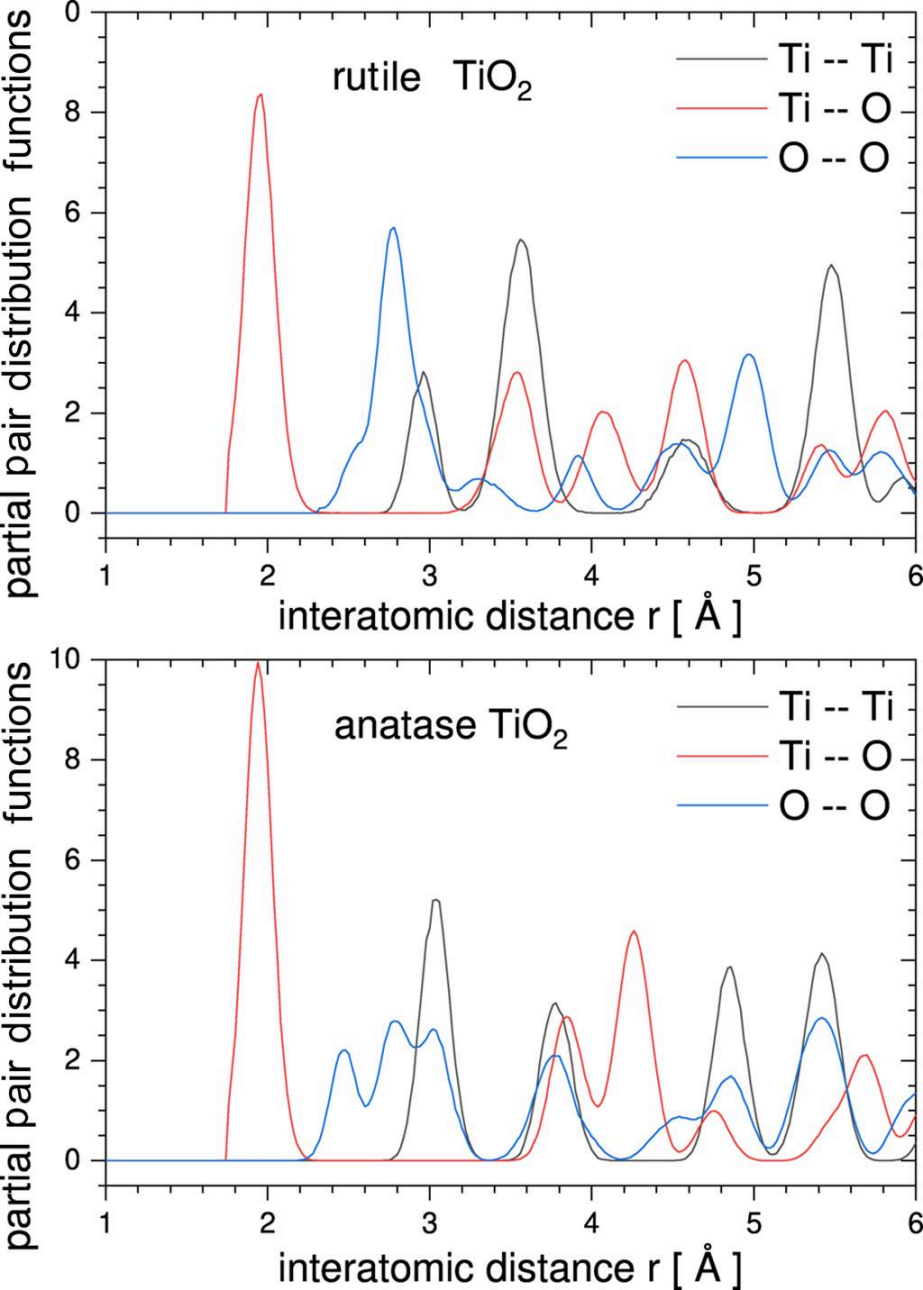
*RMCProfile7* results of partial PDFs obtained for rutile and anatase for all contributing atom pairs Ti–Ti, Ti–O and O–O.

**Figure 6:**
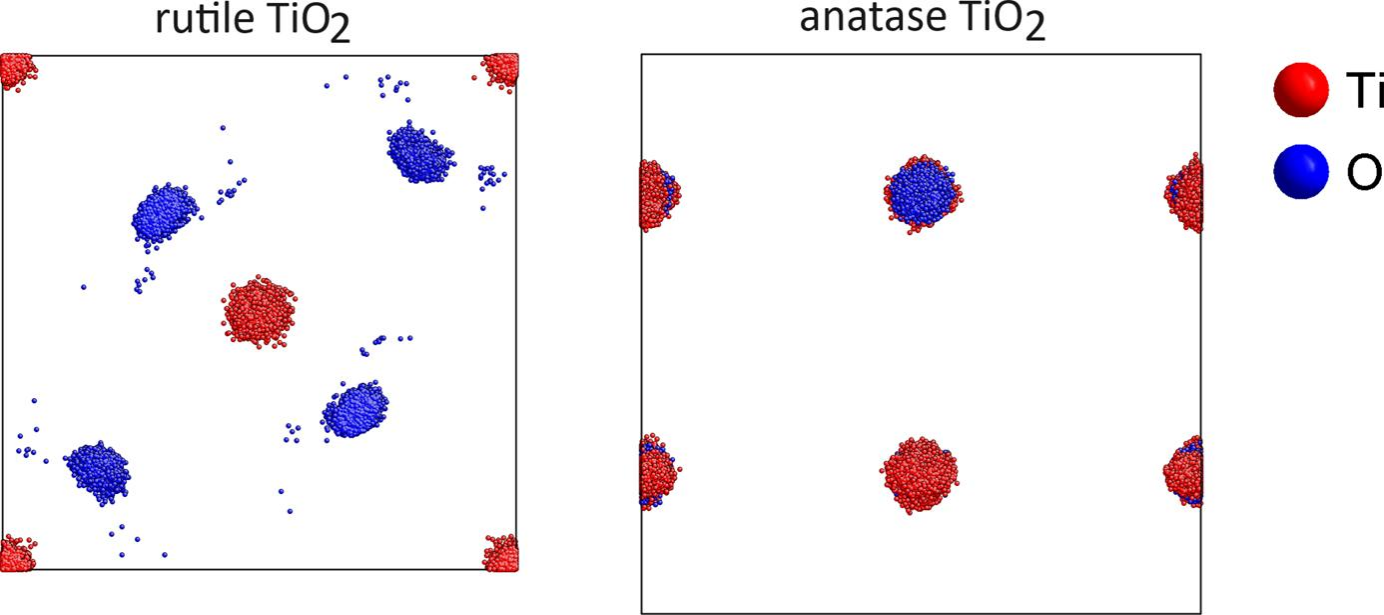
*RMCProfile7* configurations for two-phase refinement (rutile and anatase) presented as a back-projection of the supercell configuration to a single standard crystallographic unit cell.

The results of the *RMCProfile7* program multiple-phase refinements are shown in Figs. 3 ▸ and 4 ▸. The real-space *G*(*r*) and *D*(*r*), reciprocal-space structure factor *F*(*Q*), and two Bragg (bank 4 at 90° and bank 5 at 146°) average structure calculated profiles are in very good agreement with the experimental data. The ripples in the experimental structure factor *F*(*Q*) arise because the data have been convoluted with a Fourier transform of the box function. This is to account for the finite size of the configuration used for the calculation (Nield *et al.*, 1992 ▸; McGreevy, 2001 ▸). Fig. 5 ▸ shows the partial PDF obtained from the two contributing phases. Note that, even though the two structures show some similarities on the local scale, the joint refinement is sensitive enough to obtain accurate results for both phases (Fig. 6 ▸).

### 3.2. CaF_2_ minority phase refinement

**Figure 7:**
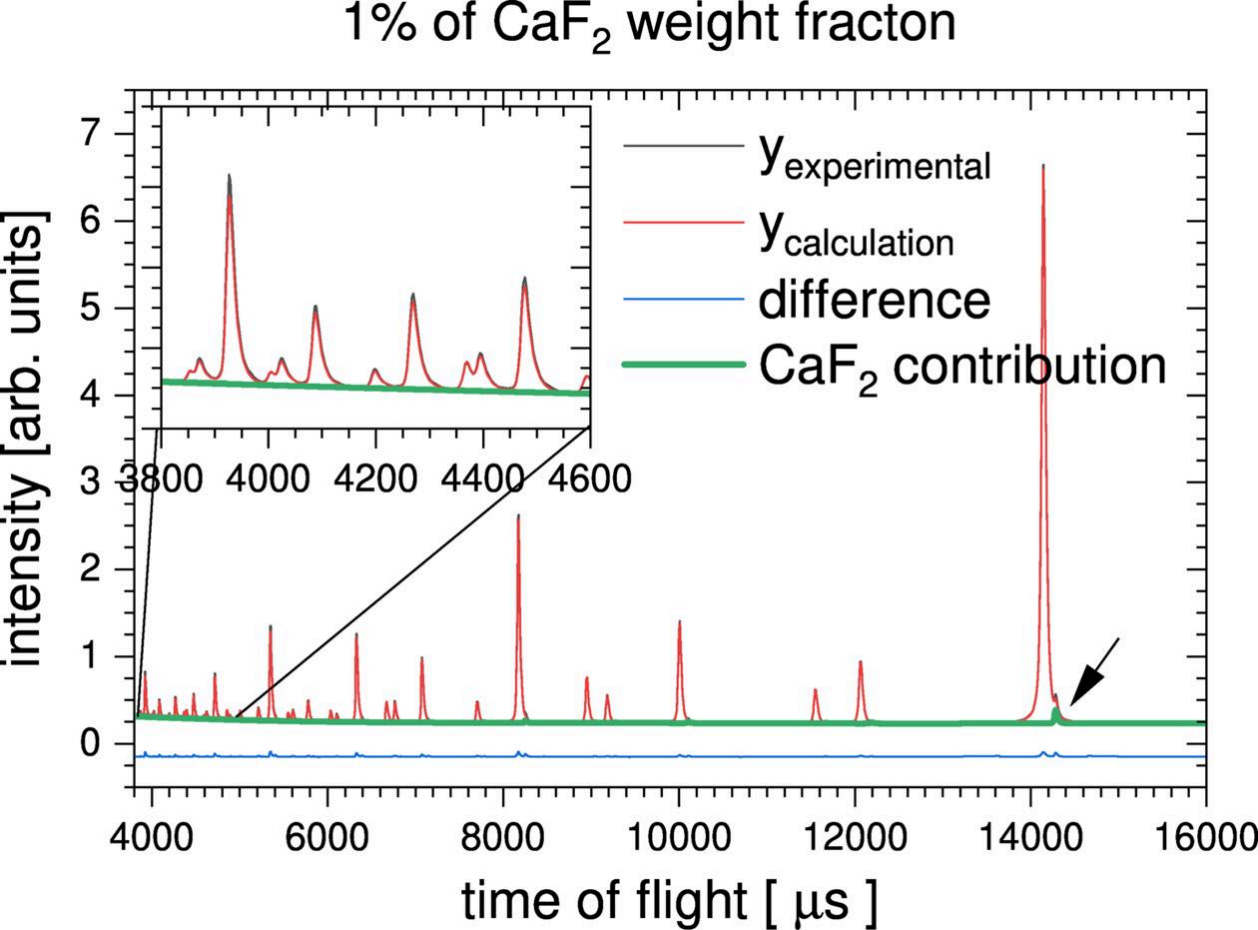
CaF_2_ minority contribution modelling in *RMCProfile7*. Simulated Bragg data set used for refinement of 1% CaF_2_ minority phase and 99% CeO_2_ (0.0218 and 0.9782 mol%, respectively). The black arrow indicates the most intense Bragg peak from the minority phase.

**Figure 8:**
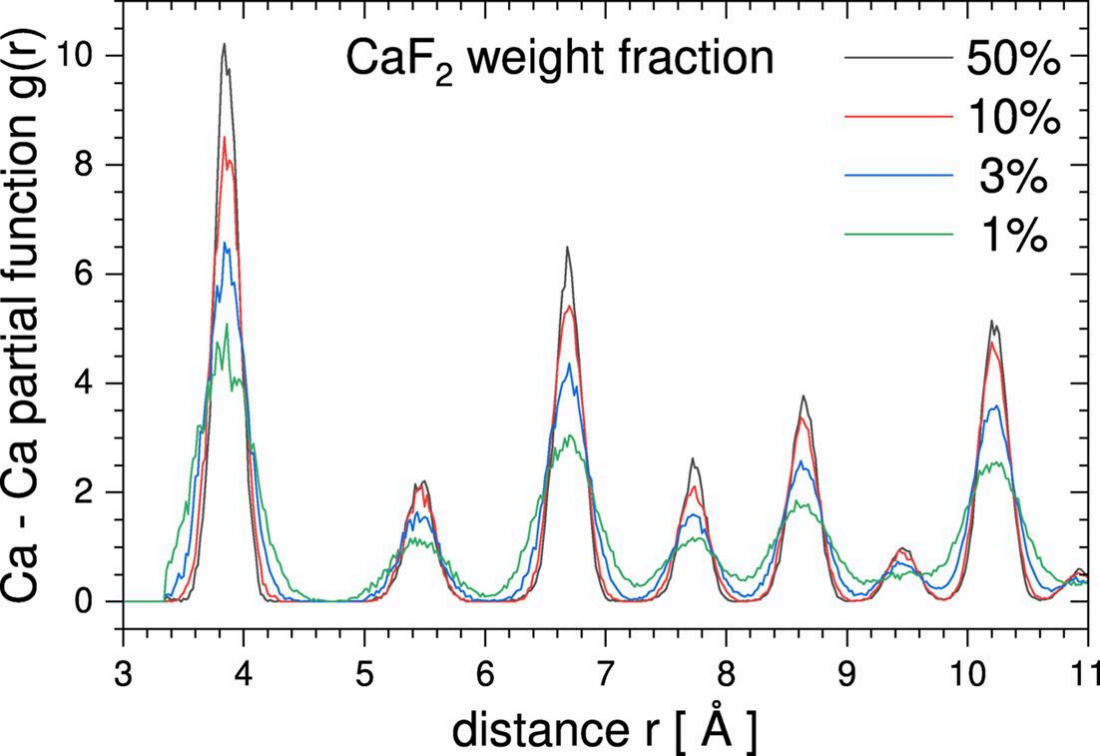
Ca–Ca partial functions obtained from simulations for the minority phase fraction from 50 to 1%.

In order to demonstrate how *RMCProfile7* can refine a secondary phase even in the case of very low phase fractions, a series of simulations has been performed for CaF_2_ and CeO_2_ mixed phases with different weight fractions. The refinement was done against simulated PDF and Bragg data sets. Fig. 7 ▸ shows the simulated Bragg time-of-flight diffraction pattern for a 1% weight fraction of CaF_2_ (0.0218 mol%) and 99% CeO_2_ (0.9782 mol%). The green line shows the minority phase contribution to the overall Bragg pattern. For 1% weight fraction (0.0218 mol%), the contribution of CaF_2_ is negligible in the low *d*-spacing region (see the inset in Fig. 7 ▸). However, even for such a small contribution to the overall diffraction data, a reasonable refinement can be obtained. In order to illustrate this, Fig. 8 ▸ shows the Ca–Ca partial function as obtained from the refinement for CaF_2_ weight fraction equal to 50, 10, 3 and 1% (0.3060, 0.1968, 0.0638 and 0.0218 mol%, respectively). In all cases the Ca–Ca partial functions have reasonable shapes, even for the lowest weight fraction. But as the minority phase contribution to the overall scattering data decreases, the width of the Ca–Ca partial functions increases.

### 3.3. Simple resolution correction applied to LiFePO_4_

**Figure 9:**
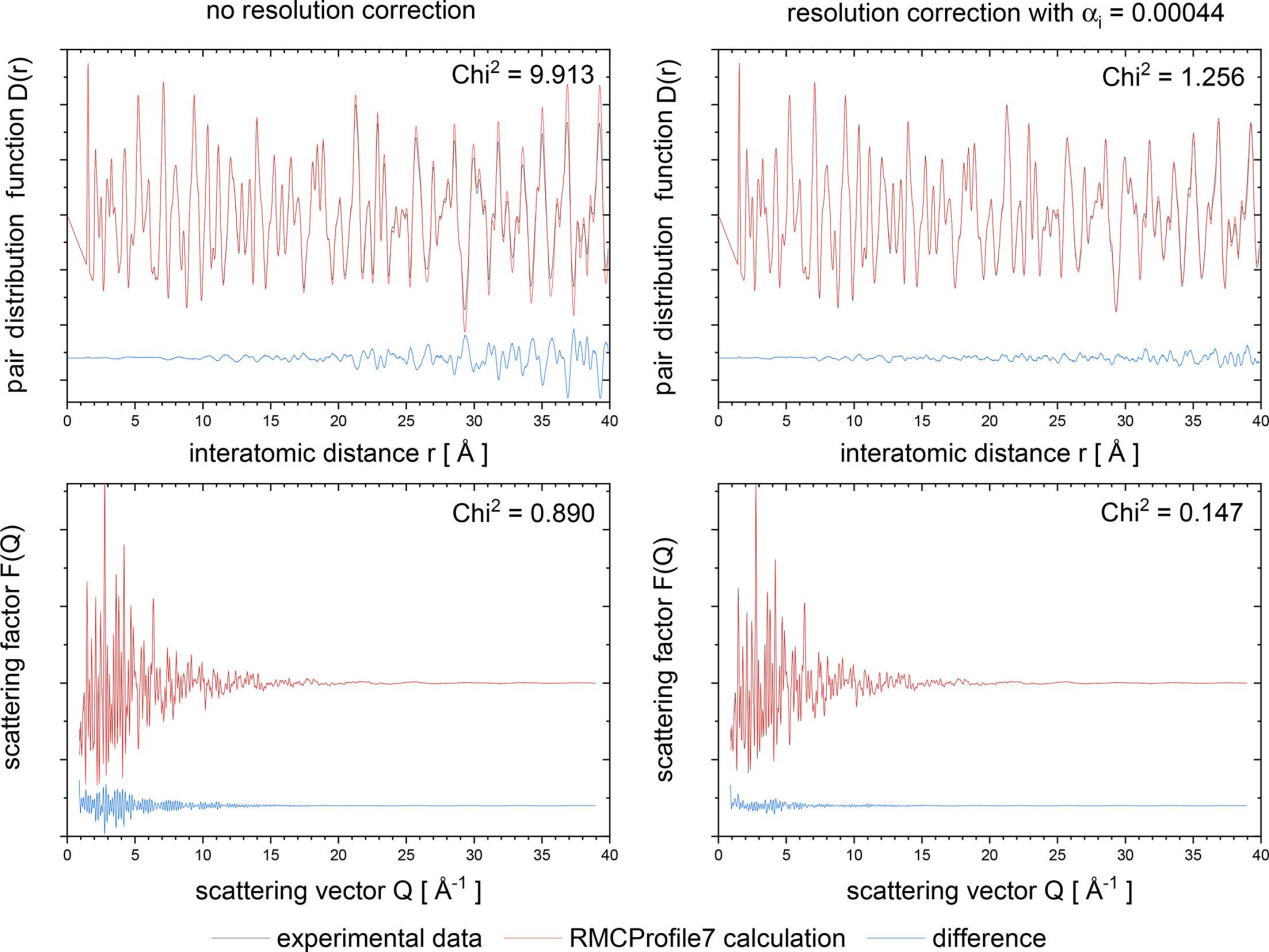
Comparison of *RMCProfile7* program results obtained without (left panels) and with (right panels) exponential resolution correction of real-space data (see text for details). Results are shown with α_*i*_ = 0.00044 Å^−2^ resolution correction applied on real-space (top panels) and reciprocal-space (bottom panels) data.

To demonstrate how the simple resolution correction (as described in Section 2.5) can improve the refinement, we have applied this correction to LiFePO_4_ data already published by Sławiński *et al.* (2019 ▸). Fig. 9 ▸ shows a comparison of the real-space (top panels) and reciprocal-space (bottom panels) data calculation of the LiFePO_4_ sample. The left panels show the results of the calculation without any resolution correction, whereas the right panels present the result of the calculation based on the same configuration but including the resolution correction, with a resolution correction coefficient α_*i*_ = 0.00044 Å^−2^. The value of α_*i*_ has been found by trial and error. One can see a significant improvement in unweighted agreement factors defined as 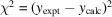 (see Fig. 9 ▸ for values).

### 3.4. Real-space PDF calculation for C_3_H_8_, CaF_2_ and NdFeO_3_

**Figure 10:**
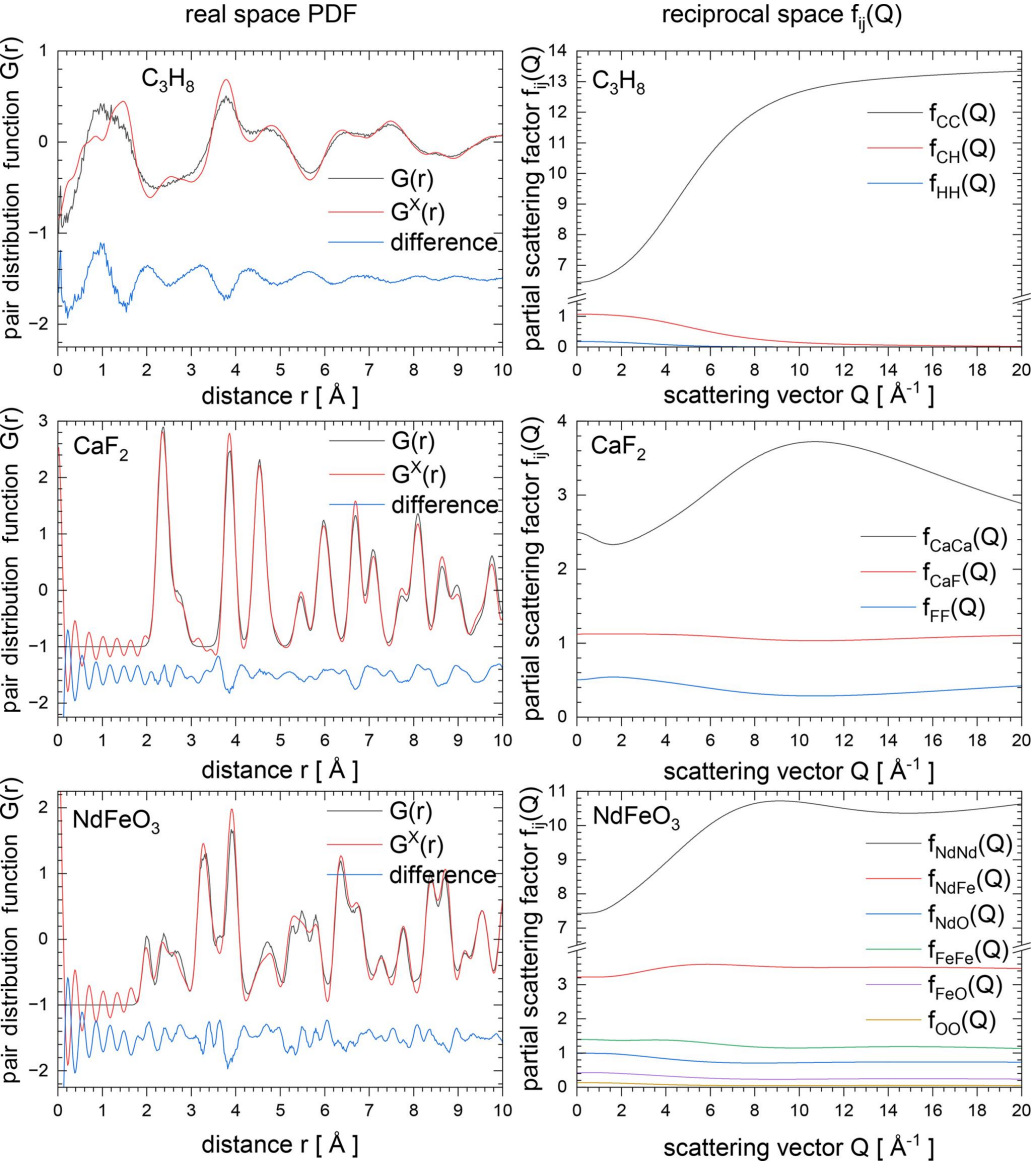
Real-space PDFs (left panels) and reciprocal-space partial form factors *f*_*ij*_(*Q*) (right panels) for C_3_H_8_, CaF_2_ and NdFeO_3_. The left panels show a comparison between PDFs calculated with histogram-based and exact methods (see text for more details).

As already explained in Section 2.6, the real-space PDF can now be calculated as a back-Fourier transform of total scattering data. Fig. 10 ▸ shows a comparison between the histogram-based [denoted as *G*(*r*)] and exact (as back-Fourier transform) calculation [denoted as *G*^X^(*r*)] of real-space data for X-rays for three examples: hydrogen-containing propene, C_3_H_8_ (Podsiadło *et al.*, 2013 ▸); comparable elements, CaF_2_ (Cheetham *et al.*, 1971 ▸); and heavy-element NdFeO_3_ (Sławiński *et al.*, 2005 ▸). Those structures have been selected to demonstrate cases where the exact X-ray PDF calculation becomes strongly recommended (light elements and materials with elements having very different atomic number). However, even for the case of CaF_2_, which contains relatively comparably weighted elements, some peak changes can be observed. The left panels show a comparison between histogram-based *G*(*r*) and exact *G*^X^(*r*) X-ray PDF calculations, whereas the right panels present X-ray partial form factors *f*_*ij*_(*Q*) used as Faber–Ziman partial weights in reciprocal space. In the case of X-ray PDF calculation, one can see not only significant broadening of selected peaks but also a serious change of peak intensity. In all cases a maximum scattering vector value *Q* equal to 20 Å^−1^ was used, the configuration sizes were approximately 80 Å along each crystallographic direction and isotropic atomic displacement parameters were used. An explicit atom distribution was calculated from the average positions.

Subsequently, the same method was used in the case of neutron scattering. Despite the scattering length being constant with *Q* for neutrons, every neutron instrument has its own limiting value of *Q*, called here the 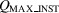 cutoff. The application of a back-Fourier transform method for the PDF calculation in the case of neutrons allows us to apply the same 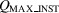 cutoff for both the data and the simulation. This results in a convolution with a 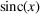 function in real space (the Fourier transform of the cutoff tophat function) and in broadening of the real-space peaks. This effect becomes stronger once the 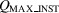 cutoff decreases and is most important for experiments with a low 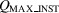 cutoff value.

**Figure 11:**
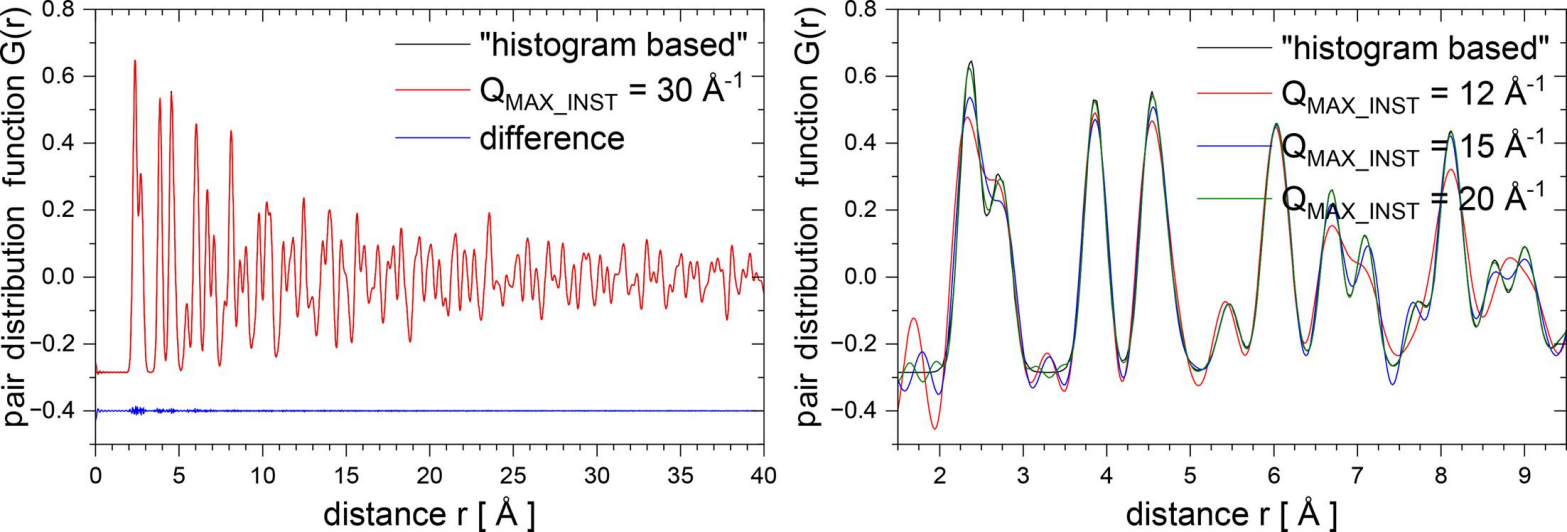
Neutron real-space PDFs calculated for CaF_2_. The left panel shows a comparison between histogram-based and 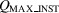 of 30 Å^−1^ PDFs (a typical value for a neutron time-of-flight experiment). The right panel shows a comparison between histogram-based and low 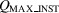 from 12 to 20 Å^−1^ PDFs.

Fig. 11 ▸ shows the results of simulations performed for CaF_2_ in the histogram-based case (used as a reference here) and for several 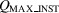 values from 12 to 30 Å^−1^. The left panel shows a comparison between histogram-based and 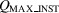 of 30 Å^−1^ PDFs (a typical value for a neutron time-of-flight experiment) where nearly no difference can be seen. However, once the value of 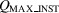 becomes lower than or equal to 20 Å^−1^, one can notice not only artificial ripples but also additional peak broadening as a result of the convolution with the sinc function, as shown in the right panel.

### 3.5. Molecular moves

In order to demonstrate how molecular moves can be used in *RMCProfile7* the SF_6_ molecular crystal has been used. The same data have already been used for structure analysis (Zhang *et al.*, 2022 ▸) and as an *RMCProfile6* tutorial. In *RMCProfile7* a general concept of a rigid-body definition is used. It allows the user to define any set of atoms to be considered as a rigid unit and called here a molecule. The following code defined the SF_6_ molecule in our example:[Chem scheme2]
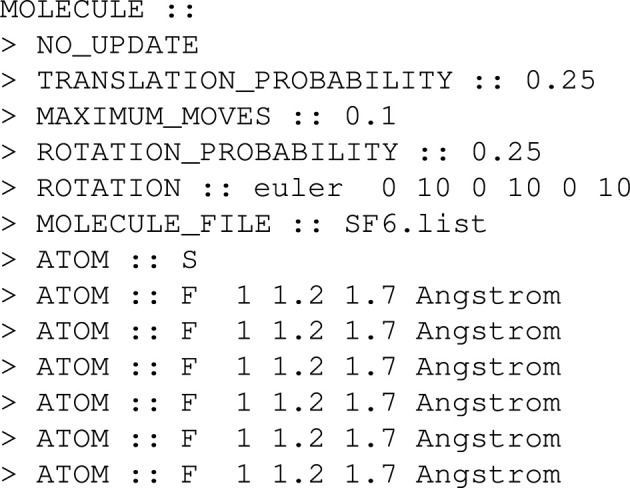


The molecule is composed of a single S atom surrounded by six F atoms. The first atom is always used as the centre and a reference point of the whole molecule. *RMCProfile7* will then search for six F atoms within the distance from 1.2 to 1.7 Å from atom number 1 (S atom). In the case that all six F atoms are found, the molecule will be stored and saved in the SF6.list file. For each molecule type a maximum move (in Å) and a minimum and maximum rotation angle can be defined. Three rotation angles are defined as Euler angles, defined as rotations along crystallographic axes: along *z*, *y*′ (the *y* axis transformed by the first rotation) and finally *z*′′ (the *z* axis transformed by the first and second rotations).

In order to illustrate rigid-body moves and their impact on the computation time, we performed two calculations using *RMCProfile7* on SF_6_ data assuming (i) 100% atomic type moves and (ii) 50% atomic type moves combined with 50% molecule (25% for translation and 25% for rotation) type moves.

**Figure 12:**
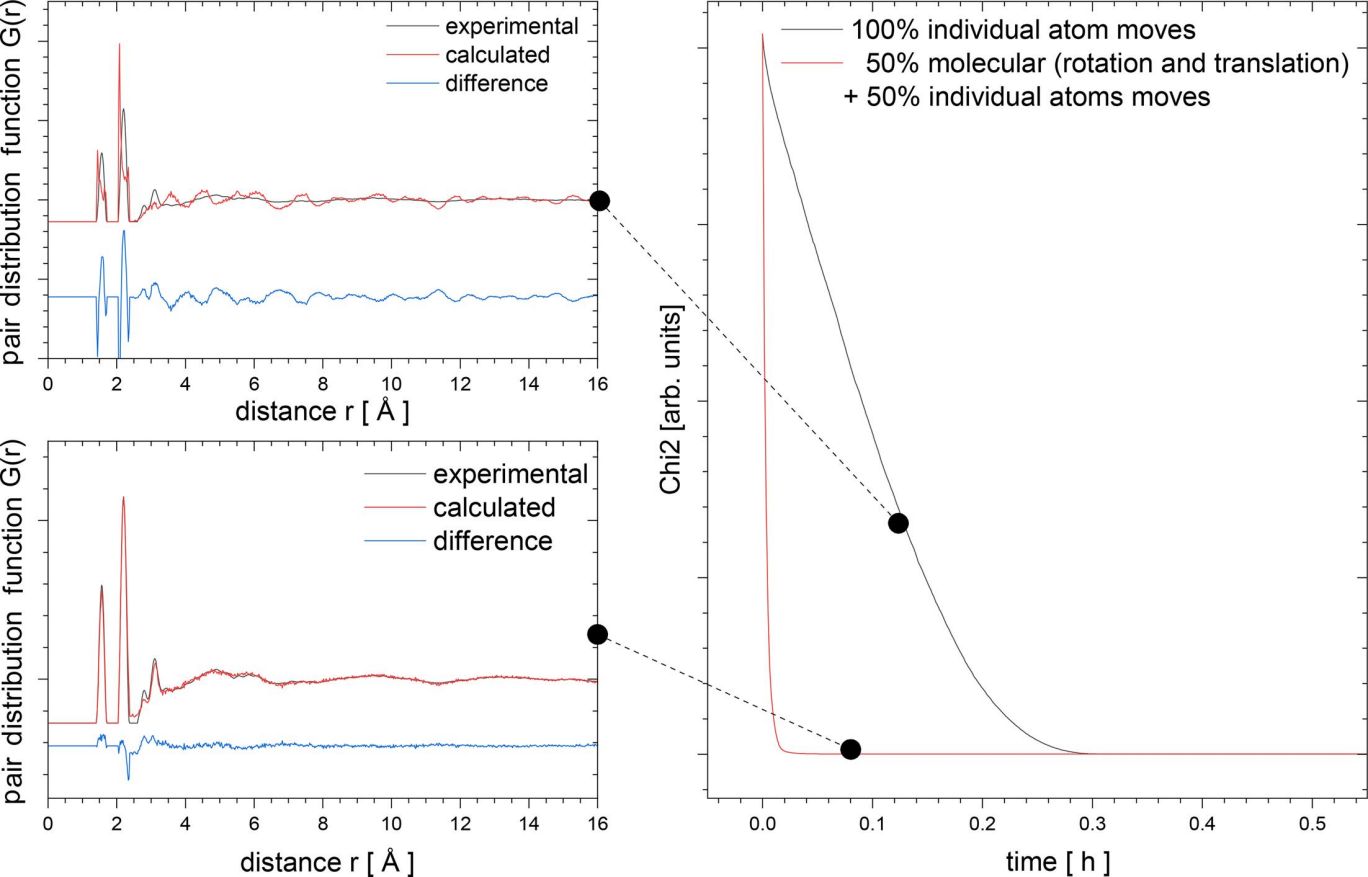
The right panel shows a comparison of overall χ^2^ agreement factor for 100% individual atom moves (black line) and a combination of 50% individual atom moves and 50% molecule type moves (translation and rotation). The two panels on the left show the refined PDF *G*(*r*) at certain times as marked by black dots.

As one can see in Fig. 12 ▸, the calculation using 50% atomic type moves combined with 50% molecule type moves converges significantly faster than that with individual moves only. The exact gain in calculation time is sample specific and cannot easily be generalized or estimated.

## 4. Summary

In this paper we introduce new, freely available software for RMC modelling of crystalline materials using total scattering data. *RMCProfile7* is the newest version of the well known *RMCProfile*. The software has already been successfully used for several scientific cases (Cai *et al.*, 2020 ▸; Nygård *et al.*, 2020 ▸, 2021 ▸) but is under constant development and support. All enquiries and questions should be addressed to Wojciech A. Sławiński (wslawinski@chem.uw.edu.pl), the main developer of the program.

## Figures and Tables

**Figure 4:**
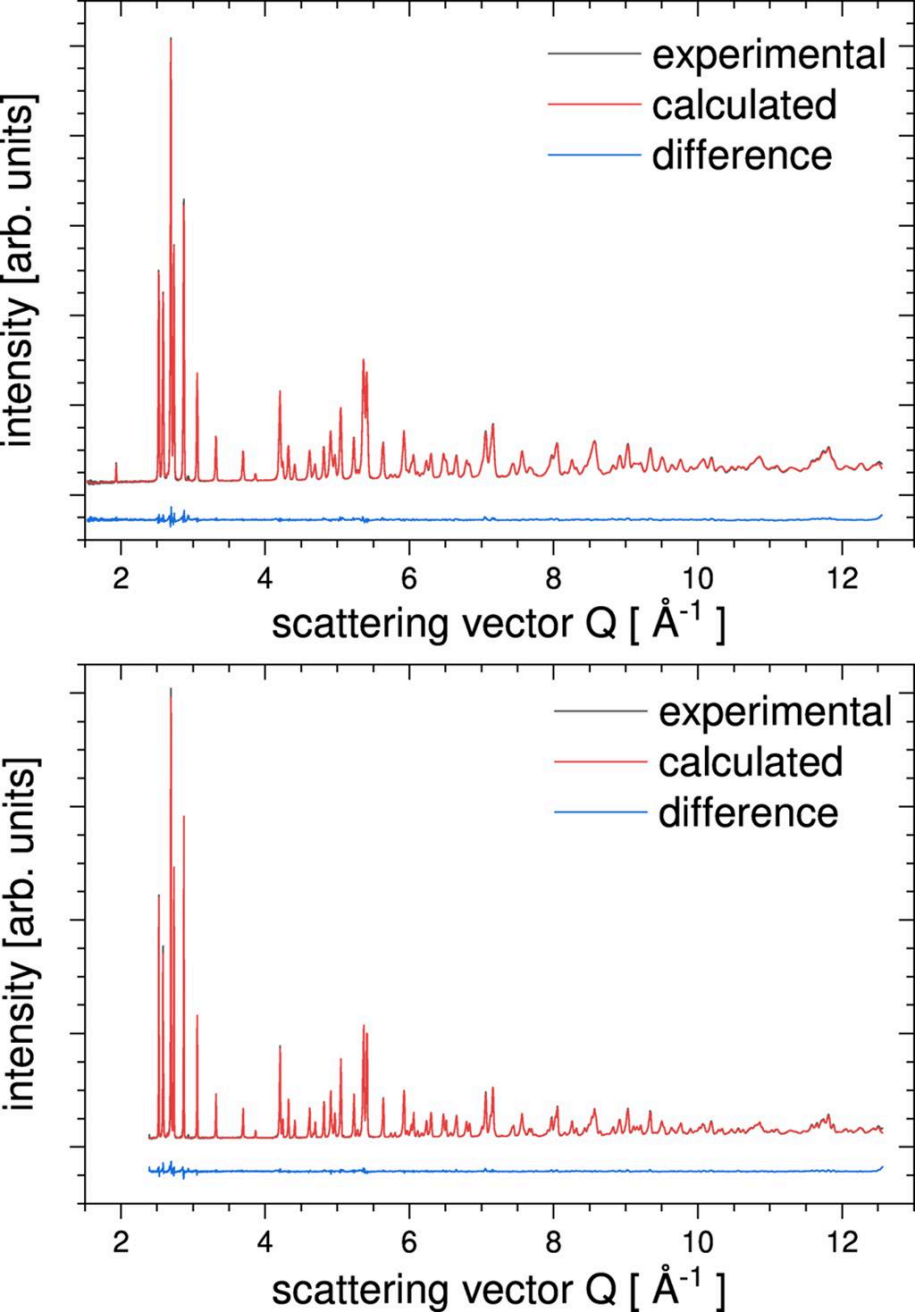
*RMCProfile7* refinements of a physical mixture 50 mol% of rutile and anatase. Experimental data, *RMCProfile7* calculation and difference curve of two Bragg data sets – bank 4 at 90° and bank 5 at 146° (top and bottom, respectively).

**Table 1:** Total scattering data set representations available in *RMCProfile7*

Data set type	*RMCProfile7* keyword	Definition
Differential correlation function	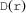	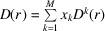
Radial distribution function	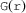	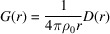
Shifted radial distribution function	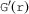	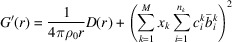
Pair distribution function (as used by *PDFgui*)	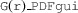	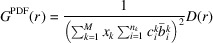
Total correlation function	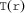	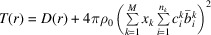
		
Total scattering function	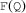	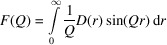
Total scattering structure factor (as used by *PDFgui*)	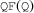	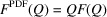
Normalized total scattering function	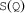	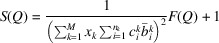

**Table 2:** Interatomic potential restraints available in *RMCProfile7*

Potential type	*RMCProfile7* keyword	Definition
Bond distance	BOND, HARMONIC	*U*(*r*_*ij*_) = *k*(*r*_*ij*_ − *r*_0_)^2^
	BOND-4-CENTRAL HARMONIC	As above, but with a specific user-defined connectivity for both *i* and *j* atoms
	BOND, MORSE	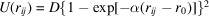
	BOND-4-CENTRAL MORSE	As above, but with a specific user-defined connectivity for both *i* and *j* atoms
		
Angle	ANGLE HARMONIC_COSINE	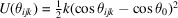
	ANGLE-NEXT-TO HARMONIC_COSINE	As above, but with a specific user-defined connectivity for the central atom
		
Dihedral angle	4-BODY, COSINE	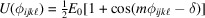
Inversion angle	4-BODY, INVERSION	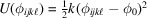
		
Planarity	PLANAR, HARMONIC	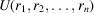 = 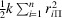 , where Π is the best-fit plane to the *n* atoms
	PLANAR-RING, HARMONIC	As above, but atoms 1 and *n* have to be connected
